# A left lung with four lobes: a new discovery during the thoracotomy for recurrent primary spontaneous pneumothorax

**DOI:** 10.1186/s13019-021-01651-3

**Published:** 2021-09-28

**Authors:** Saadat Mehrabi, Nader Tanideh, Reza Hosseinpour, Cambyz Irajie, Mohammad Javad Yavari Barhaghtalabi

**Affiliations:** 1 Department of General Surgery, Shahid Beheshti Hospital, Yasuj University of Medical Sciences, Yasuj, Iran; 2 Stem Cells Technology Research Center, Stem Cells Research Institute, Shiraz University of Medical Sciences, Shiraz, Iran; 3 Department of Medical Biotechnology, School of Advanced Medical Sciences and Technologies, Shiraz University of Medical Sciences, Shiraz, Iran

**Keywords:** Primary spontaneous pneumothorax, Left lung, Thoracotomy, Four lobes, New discovery

## Abstract

**Background:**

The right and left lung anatomy are similar but asymmetrical. The right lung consists of three lobes, and the left lung consists of two lobes. Our study is unique because of discovering a very rare morphological feature of the left lung which has not been reported yet. By the way, we compared two different available chemical agents for pleurodesis (talc and bleomycin) according to side effects, complications, and pneumothorax recurrence.

**Case presentation:**

We reported a case of bilateral primary spontaneous pneumothorax, who underwent talc slurry and bleomycin pleurodesis at right and left side retrospectively, and then complicate with left-sided recurrent spontaneous pneumothorax, so underwent open thoracotomy and was surprisingly and accidentally found to have 4 lobes and 3 fissures in left lung.

**Conclusion:**

In our case report, there were one main oblique fissure and two accessory fissures which divided the lung into 4 separated lobes, and this discovery in human’s and other animals’ lung anatomy has not been previously reported. In our case study, the talc slurry was more effective in preventing spontaneous pneumothorax recurrence, but with more side effects than bleomycin. We could hypothesize that the morphological variation of the lung might affect spontaneous pneumothorax development and recurrence.

## Introduction

The right and left lung anatomy are similar but asymmetrical. The right lung consists of three lobes: the right upper lobe (RUL), the right middle lobe (RML), and the right lower lobe (RLL). The right lobe is divided by an oblique and horizontal fissure, where the horizontal fissure divides the upper and middle lobe, and the oblique fissure divides the middle and lower lobes. The left lung consists of two lobes: the left upper lobe (LUL) and the left lower lobe (LLL). In the left lobe, there is only an oblique fissure that separates the upper and lower lobe [1].

Lobation varies greatly among species. Table 1 compares the lung lobes in different species of animals [2–11].

**Table 1:** Interspecies comparison of Lung lobes

	Number of right lung lobes	Description of right lung lobes	Number of left lung lobes	Description of left lung lobes
Human (1, 2)	3	–	2	–
Dog (2, 3)	4	Cranial, middle, caudal, and accessory lobes	2 or 3	Apical, middle, and diaphragmatic lobes, left apical and middle lobes are partially fused, being separated only by an incomplete (cranial) fissure
Cat (2, 4)	4	Cranial, middle, caudal and accessory lobes	2 or 3	Apical (cranial), middle (cardiac), and diaphragmatic (caudal) lobes (3 lobes theory) versus apical (cranial) and diaphragmatic (caudal) lobes (2 lobes theory)
Bovine (5)	4	Cranial, middle, caudal and accessory lobes	2	Cranial and caudal lobes
Horse (2, 6)	1	Cranial, middle, caudal and accessory lobular divisions are united to form 1 lobe	1	Cranial, middle, caudal and accessory lobular divisions are united to form 1 lobe
Raccoon (2)	4	–	3	–
Equine (7)	3	cranial, caudal and accessory lobes	2	Cranial and caudal lobes
Mouse (2, 9, 10)	4	Cranial, middle, caudal, and accessory lobes	1	No division
Rat (2, 10, 11)	4	Upper, middle, accessory and lower lobes	1	The upper and accessory lobes are lost. The middle and lower lobes are united to form one lobe
Hamster (11)	5	cranial, middle, caudal, intermediate, accessory	1	Single lobe

Spontaneous pneumothorax occurs secondary to intrinsic abnormalities of the lung without any preceding trauma or obvious precipitating causes, and can be classified as primary and secondary. Primary spontaneous pneumothorax is defined as a spontaneous pneumothorax without underlying lung disease, while secondary spontaneous pneumothorax refers to those that develop in the presence of an underlying lung disease such as emphysema (rupture of a bleb or bulla), cystic fibrosis, acquired immunodeficiency syndrome (AIDS), metastatic cancer (especially sarcoma), asthma, lung abscess, occasionally lung cancer, and Catamenial pneumothorax [12, 13]. Apical subpleural bleb rupture is the most common cause. The cause of these blebs is unknown, but they occur more frequently in smokers and young post-adolescent males with a tall thin body habitus. Treatment is generally chest tube insertion with water seal [12].

It has been demonstrated that 32% of patients will develop a recurrence, with most of the risk in the first year. Recurrence rates did not differ based on the initial intervention for primary spontaneous pneumothorax. Female sex, lower body mass index (BMI), smoking cessation and radiological evidence of dystrophic lung lesions (evaluated with computed tomography (CT) scan according to the pulmonary dystrophic lesions score (number and size of blebs and bullae)), are factors which are associated with a higher risk of recurrence [14, 15].

Other than emptying air from the pleural cavity by simple aspiration or chest tube drainage, the management of spontaneous pneumothorax also focused on the stopping of the air leak and avoiding the recurrence by surgical intervention or chemical pleurodesis. Making symphysis between the two layers of pleura by sclerosing agents is called chemical pleurodesis. Sclerosing agents which could be used for chemical pleurodesis are talc, tetracycline, minocycline, bleomycin, autologous blood patch, OK-432 (Picibanil), and Iodopovidone [16].

Surgery is specified to disease process and may involve surgical pleurectomy, pleurodesis, bullectomy, and lung resection through video-assisted thoracoscopic surgery (VATS), axillary mini-thoracotomy (AMT) and formal anterolateral or posterolateral thoracotomy [12, 17].

In this study, we reported a case of bilateral primary spontaneous pneumothorax, who underwent talc slurry and bleomycin pleurodesis at right and left side respectively, and then complicated with left sided recurrent spontaneous pneumothorax, so underwent open thoracotomy and was surprisingly and accidentally found to have 4 lobes and 3 fissures in left lung. Our study is unique because of discovering a very rare morphological feature of left lung which has not been reported yet. By the way, we compared two different available chemical agents for pleurodesis (talc and bleomycin) according to side effects, complications, and pneumothorax recurrence.

## Case presentation

**Fig. 1:**
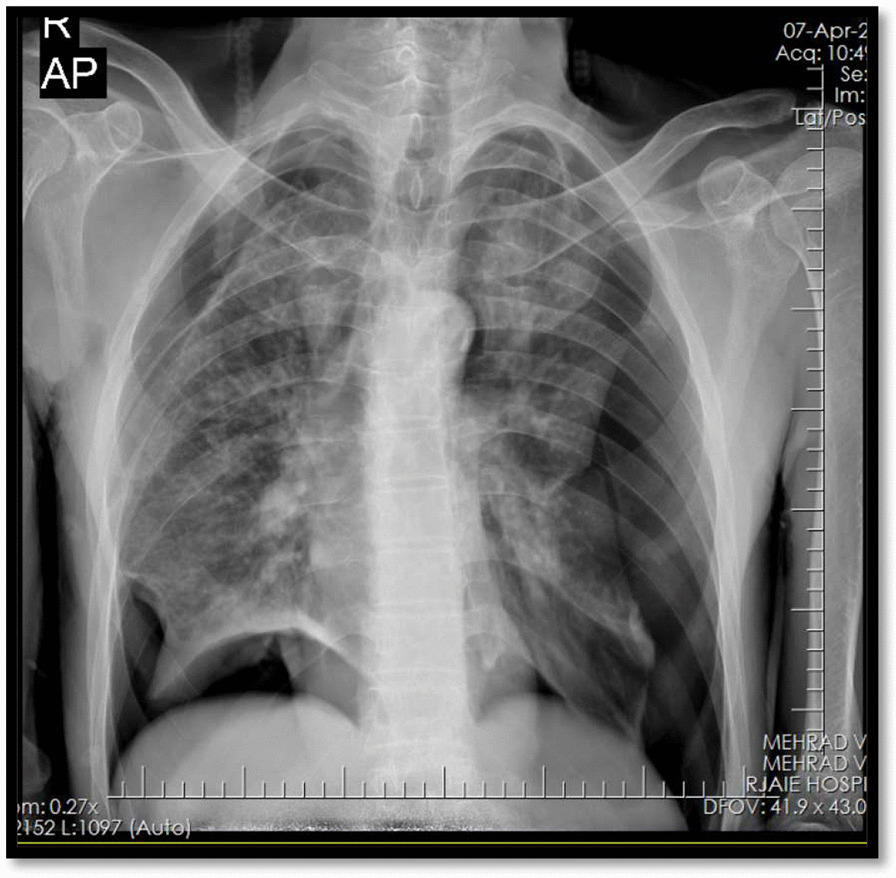
Chest radiography (1st admission), bilateral primary spontaneous pneumothorax

**Fig. 2:**
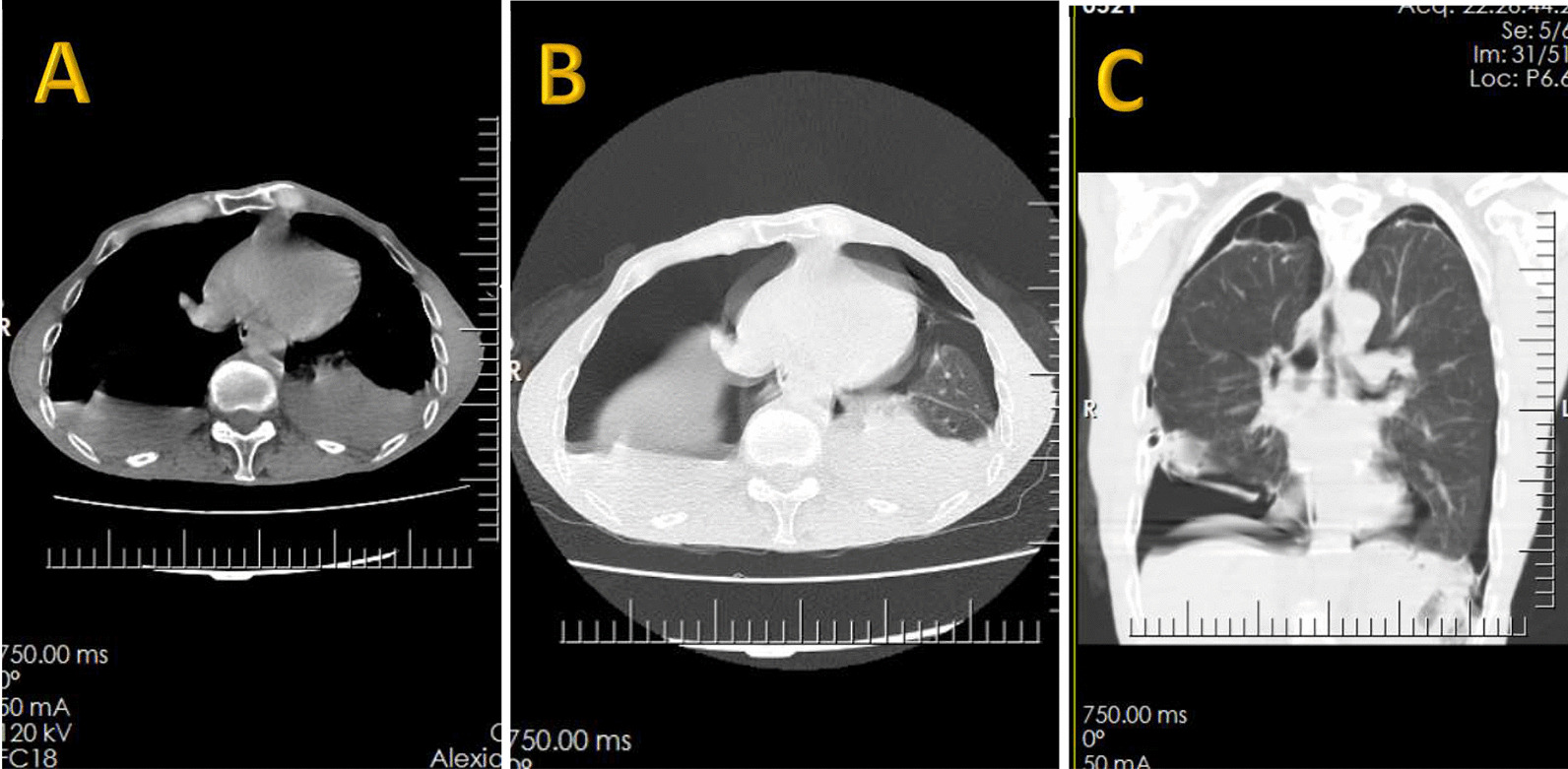
Spiral chest CT scan without contrast (1st admission), bilateral primary spontaneous pneumothorax, **a** mediastinal axial view, **b** lung window axial view, **c** lung window coronal view

**Fig. 3:**
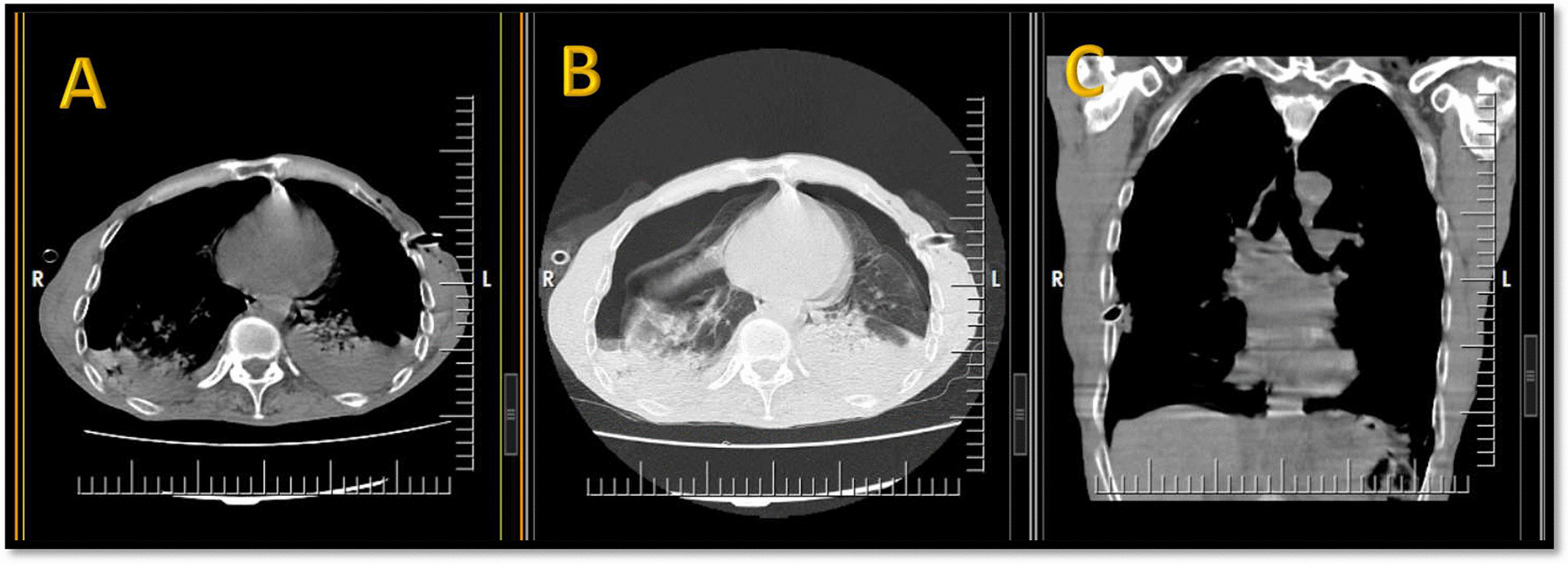
Spiral chest CT scan without contrast (1st admission), inserted bilateral chest tubes, **a** mediastinal axial view, **b** lung window axial view, **c** mediastinal coronal view

The patient was a 47-year-old man who was brought to the emergency department of Shahid Beheshti hospital with chief complaints of dyspnea and dry cough but no respiratory distress for two days before the admission. The patient had an amputated left leg and knee due to the previous trauma since childhood and was a heavy smoker (1 pack-year) and had inhalational opium addiction for more than 20 and 10 years respectively. The patient had no family history of any specific disease. The patient’s upright chest radiography and Chest spiral CT scan revealed bilateral primary spontaneous pneumothorax (Figs. 1, 2), and the patient underwent bilateral chest tube insertion (After local anesthesia, a conventional large-bore chest tube (32 French) was placed into the pleural space in the bilateral 5th-6th intercostal space in the posterior axillary line (Fig. 3).

**Fig. 4:**
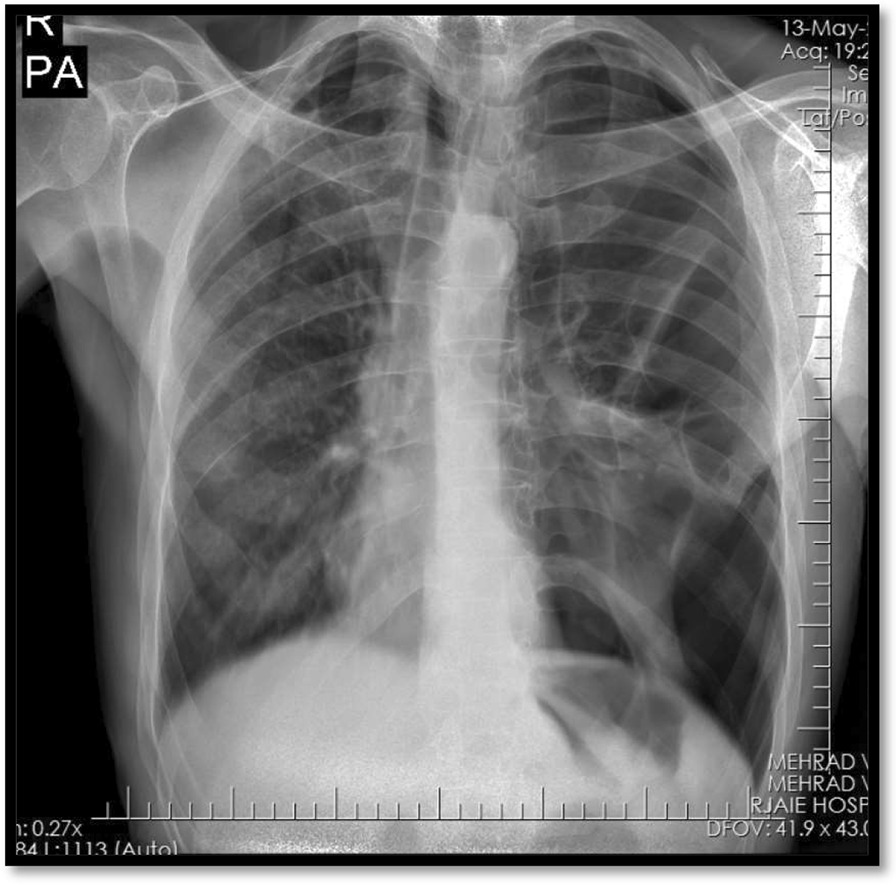
Chest radiography (2nd admission), left sided recurrent spontaneous pneumothorax

**Fig. 5:**
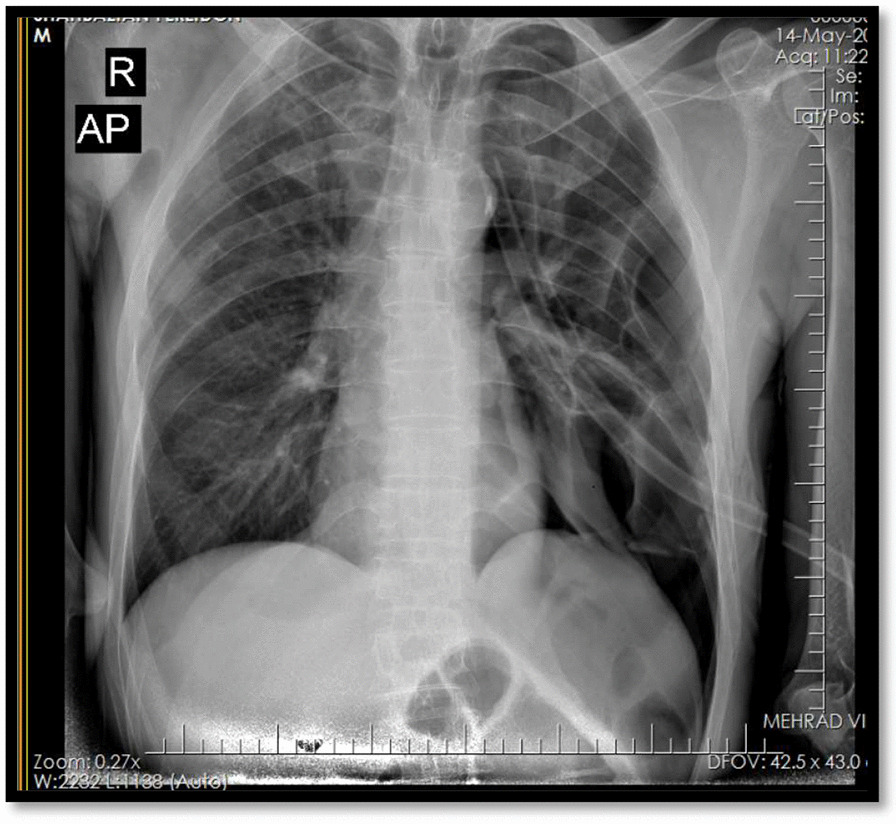
Chest radiography (2nd admission), not-resolved left sided pneumothorax after the chest tube insertion

**Fig. 6:**
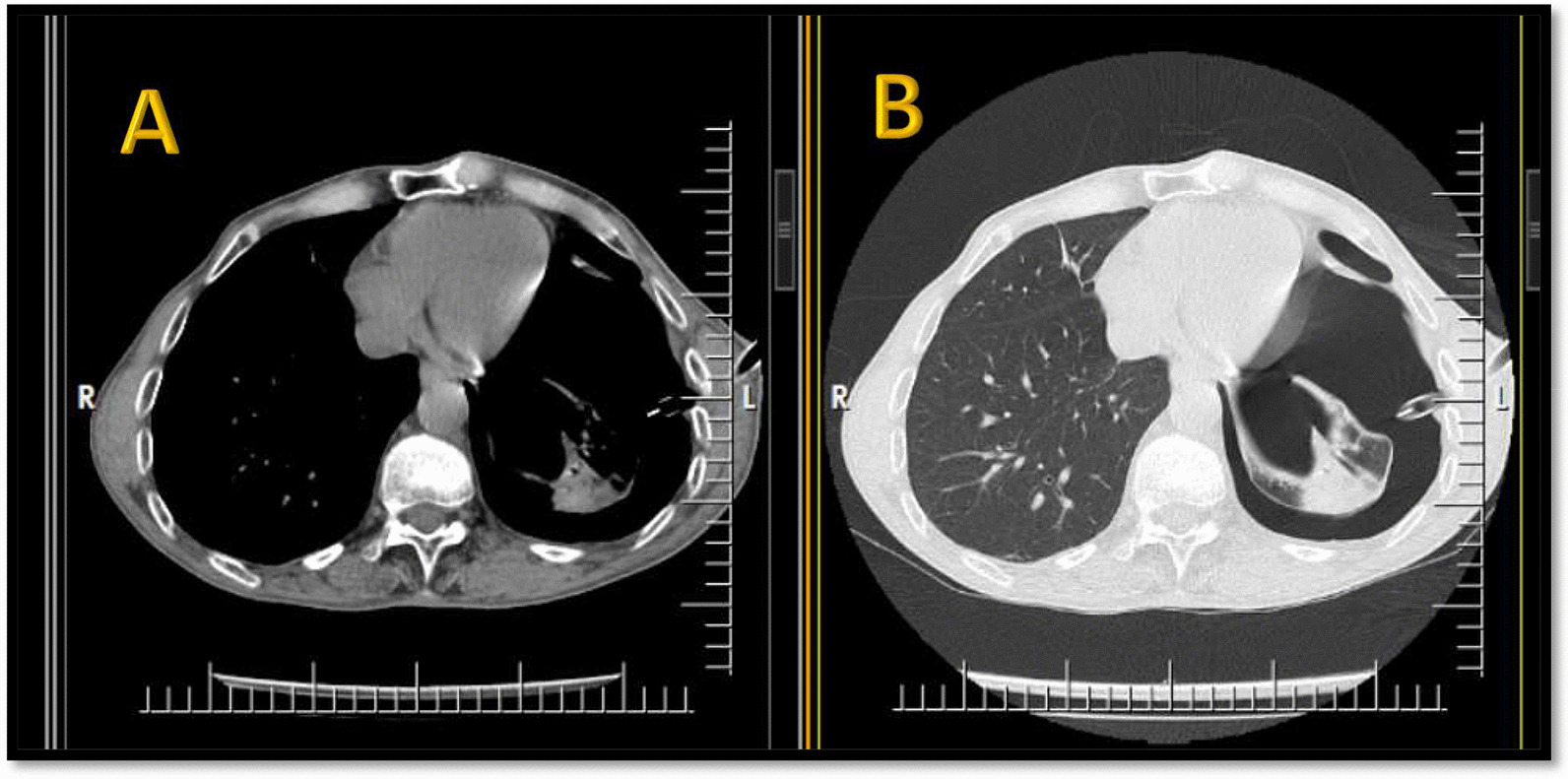
Spiral chest CT scan without contrast (2nd admission), not-resolved left sided pneumothorax after the chest tube insertion, **a** mediastinal axial view, **b** lung window axial view

Two days after the insertion of the chest tubes, as there was no development of bullae within the lung parenchyma as the bullous lung disease, and after obtaining an informed consent from the patient, the patient underwent chemical pleurodesis with talc slurry at the right side, and on the next day chemical pleurodesis with bleomycin was done at the left side (Bilateral pleurodesis was done in two consecutive days). For talc slurry and bleomycin pleurodesis, 2 g of talc and 60 units of bleomycin were dissolved in 50 ml sodium chloride 0.9% respectively and were shacked to ensure the thorough mixing before flushing it with a 50 ml syringe into the chest tube and then the intercostal tube was clamped for 4 to 6 h. During this time, the patient was told to rotate to prone supine and left and right lateral decubitus positions for every 20 min to make sure good spread of the talc slurry or bleomycin in the pleural cavity, and then the chest tube was unclamped, and was removed within 48 h. The patient received heart monitoring and pulse oximetry during and after the administration of talc or bleomycin until the chest tube was removed. The patient was discharged and was followed afterward. He developed again with severe dyspnea and respiratory distress 14 month after the first presentation of the disease, so was admitted and the patient’s upright chest radiography revealed recurrent left-sided spontaneous pneumothorax (Fig. 4). In Table 2, comparing of talc slurry and bleomycin pleurodesis is shown according to the side effects, complications, and pneumothorax recurrence. Left sided chest tube was inserted again for him, but unfortunately the lung was not expanded afterward, moreover, the patient intervention adherence and tolerability in using incentive spirometry was very poor, so the patient was candidate for surgery. Figures 5 and 6 show that the pneumothorax didn’t resolve.

**Table 2:** Comparing of talc slurry and bleomycin according to side effects, complications, and pneumothorax recurrence

Effect on pneumothorax, Side Effects, complications, and recurrence	Talc, right side	Bleomycin, left side
Pain (pleuritic chest pain)	++	+
Nausea	+	−
Fever	++	+
Tachycardia	+	−
Hypotension	−	−
Breathlessness and dyspnea	+	−
Cough	+	−
Respiratory distress	−	−
Decreased O2 saturation	−	−
Hypersensitivity to the drug	−	−
Recurrent pneumothorax	−	+
Pethidine use	++	+
Pleural effusion	−	−
Pneumonia	−	−
Hemothorax	−	−
Pneumothorax recurrence	−	+

**Fig. 7:**
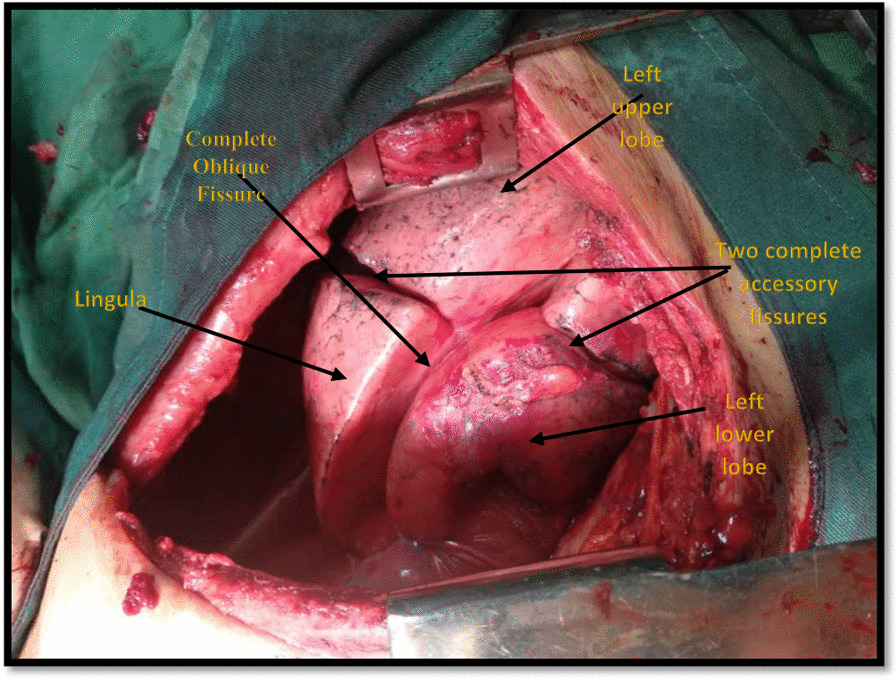
Left posterolateral thoracotomy, A left lung with 4 lobes and 3 developed fissures

**Fig. 8:**
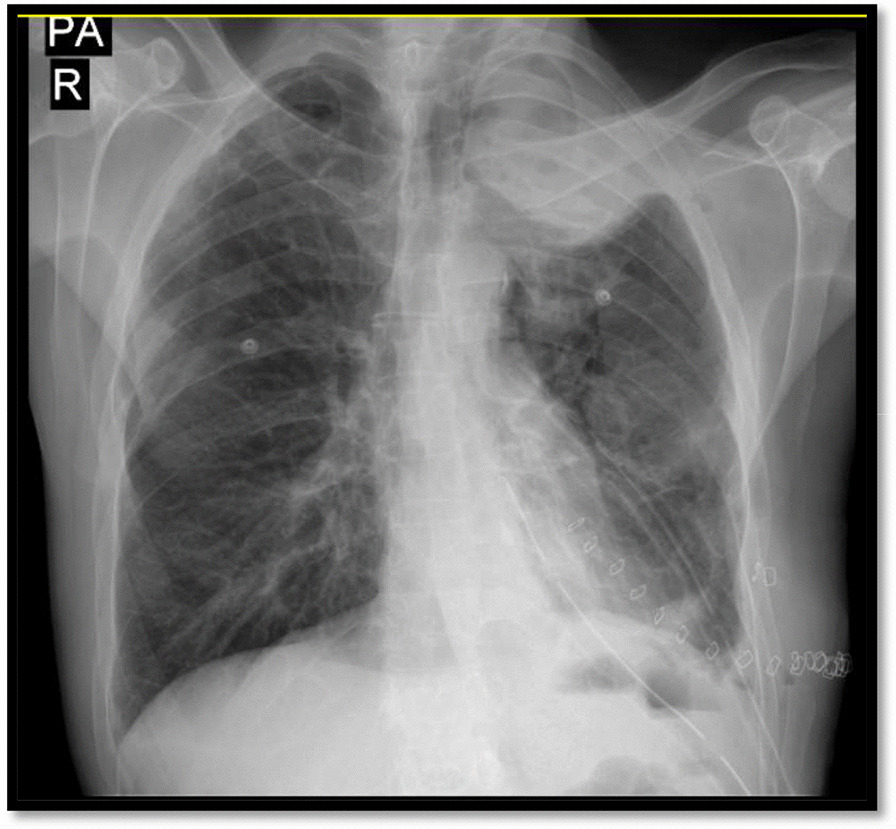
Chest radiography (2nd admission), after the operation showing two chest tubes, and resolved pneumothorax

The patient underwent posterolateral thoracotomy, pneumolysis, and wedge resection of apical segment of left upper lobe (bullectomy), apical pleurectomy, and scarification. We found an accidental and surprising finding in left lung during thoracotomy and that was that the left lung had 4 lobes with 3 developed fissures (Fig. 7). Figure 8 shows chest radiography after the operation.

**Fig. 9:**
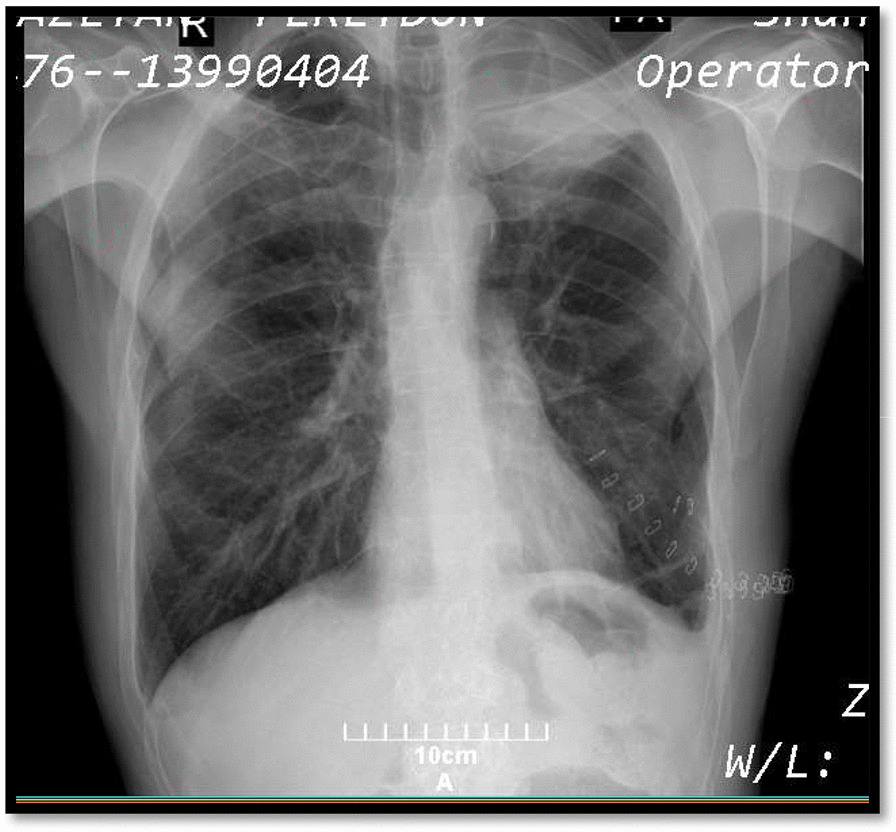
Chest radiography (2nd admission), at the time of discharge from the hospital showing discontinued chest tube, and resolved pneumothorax

The patient was then discharged with a good recovery after 7 days. Figure 9 shows the chest radiography on the time of discharge from the hospital. There was no report of the recurrence afterward in follow-up visits.

## Discussion

In our study, using talc suspension (slurry) was resulted in pain (pleuritic chest pain), nausea, fever, tachycardia, breathlessness or dyspnea, and cough; and using bleomycin was resulted in pain (pleuritic chest pain), fever, and recurrent spontaneous pneumothorax. Fever and pain were the side effects of pleurodesis seen in usage of both talc and bleomycin. Pethidine was used more often in talc than the bleomycin pleurodesis.

Our results were compatible with the results of other studies according to the talc effects [16, 18]. Talc poudrage and slurry were supposed to be more effective, but were associated with more complications, including respiratory failure [18]. There are at least 32 cases in the literature in whom acute respiratory distress syndrome was developed after administration of talc [19], but in our study, the patient didn’t develop respiratory distress after talc pleurodesis.

An animal model showed that the intrapleural injection of bleomycin was ineffective in creating pleural fibrosis, and that the bleomycin is expensive and relatively ineffective compared with other sclerosing agents; therefore, it is not suggested to be used as a pleural sclerosant in patients with non-neoplastic pleural disease (like pneumothorax) [16, 20, 21]. Our results for the administration of bleomycin was compatible with the results from other studies mentioned above, as the pneumothorax occurred again at the side in which bleomycin was administered before.

Upper and a lower lobes divide the left lung by the means of the oblique fissure [1, 22]. The left lung does not have a middle lobe in contrast to the right lung, however it does have a projection of the upper lobe called lingula. The lingula on the left lung is as the same as the middle lobe in the right lung. In a study done on the Nepalese cadavers showed two left lungs with lingula appearing as a separate lobe [22]. In another study done on the Indian cadavers showed that from 73 left lungs, there were two (2.73%) lungs with 2 fissures (one of them considered to be an accessary fissure) and they divided the left lung in to 3 lobes instead of 2 lobes [23], and this result shows that just the three lobes in the left lung has the highest number of lobes which has been reported in the literature in the human studies. In the other animals, as was described in Table 1, no separated 4 lobes have been identified. In our case report, there were one main oblique fissure and two accessory fissures which divided the lung into 4 separated lobes, and this discovery in human’s and other animals’ lung anatomy has not been previously reported. (Fig. 6).

## Conclusion

In our case study, the talc slurry was more effective in preventing spontaneous pneumothorax recurrence, but with more side effects than bleomycin. We could hypothesize that the morphological variation of the lung might affect spontaneous pneumothorax development and recurrence. Variations in the number and pattern of lobes in both human lungs is obscure, and there is little knowledge about the association of some lung diseases like spontaneous pneumothorax with these morphological variations, so some relevant future studies are needed to prove this claim.

## Data Availability

The datasets used and/or analyzed during the current study are available from the corresponding author on reasonable request.

## Abbreviations

RUL: Right upper lobe
RML: Right middle lobe
RLL: Right lower lobe
LUL: Left upper lobe
LLL: Left lower lobe
CT scan: Computed Tomography scan
